# Implicit, Explicit, and Structural Barriers and Facilitators for Information and Communication Technology Access in Older Adults

**DOI:** 10.3389/fpsyg.2022.874025

**Published:** 2022-05-17

**Authors:** Miguel Gomez-Hernandez, Stine Willum Adrian, Xavier Ferre, Elena Villalba-Mora

**Affiliations:** ^1^Ageing Lab, Center for Biomedical Technology, Universidad Politécnica de Madrid, Madrid, Spain; ^2^Department of Learning and Philosophy, Aalborg University, Copenhagen, Denmark

**Keywords:** difficulty, technology use, ethnography, motivation, social identities, user experience, COVID-19, politic and cultural process

## Abstract

Older adults’ usage of information and communication technology (ICT) is challenged or facilitated by perception of usefulness, technology design, gender, social class, and other unspoken and political elements. However, studies on the use of ICT by older adults have traditionally focused on explicit interactions (e.g., usability). The article then analyzes how symbolic, institutional, and material elements enable or hinder older adults from using ICT. Our ethnographic methodology includes several techniques with Spanish older adults: 15 semi-structured interviews, participant observation in nine ICT classes, online participant observation on WhatsApp and Jitsi for 3 months, and nine phone interviews due to COVID-19. The qualitative data were analyzed through Situational Analysis. We find that the elements hindering or facilitating ICT practice are implicit-symbolic (children’s surveillance, paternalism, fear, optimism, low self-esteem, and contradictory speech-act), explicit-material (affordances, physical limitations, and motivations), and structural-political (management, the pandemic, teaching, and media skepticism). Furthermore, unprivileged identities hampered the ICT practices: female gender, blue-collar jobs, illiteracy, and elementary education. However, being motivated to use ICT prevailed over having unprivileged identities. The study concludes that society and researchers should perceive older adults as operative with technologies and examine beyond explicit elements. We urge exploration of how older adults’ social identities and how situatedness affects ICT practice. Concerning explicit elements, Spanish authorities should improve and adapt ICT facilities at public senior centers and older adults’ homes, and ICT courses should foster tablet and smartphone training over computers.

## Introduction

*He –my husband– does not let me touch the cellphone as I damage it. He claims that I bust everything I touch so I get afraid of it!* (Natalia, old woman, 88 years old).

An older adult couple over 65 years old talks about their life’s stories, troubles, reasons, and expectations embodying their experiences with mobile phones. Natalia does not feel confident with the device since her husband is responsible for the devices at home but does not help her learn to use them. This quote represents what discrimination in technology entails: it is not only related to age but also other social identities, like gender.

This study focuses on aging and ICT because we expect the older Spanish population to grow from 19.2 to 25.2% by 2033 (INE, 2018a). Moreover, we face a large digital divide in Spain: 36 percent of people 65–74 years old do not use the internet, while 99.5 percent between 16 and 24 years old do (Pérez Díaz et al., 2020:37).

The article broadly aims to unpack what enables or hinders Spanish older adults from using ICT. Our method is qualitative and inductive to acknowledge older adults’ diverse social backgrounds. By exploring this, we employ three analytical frameworks: explicit, implicit, and structural barriers or facilitators. These frameworks are in a way inspired by other studies, but the concepts together have not yet been used in the field of aging and technology. The explicit, implicit, and structural frameworks should not be understood as micro–meso–macro in that our frameworks are not hierarchical and the conceptualization emerges, namely from our interpretation of the data of this research. We interpreted the data through a Situational Analysis suggested by Clarke and Friese (2007) to identify and represent the complexity and variation of data, and reveal hidden perspectives. This type of analysis uncovered the implicit and structural frameworks, beyond explicit elements.

The explicit framework examines the bodily and material experiences of older adults practicing ICT, i.e., what is easily observable such as a physical barrier or a clear preference for technology. For example, Gitlow (2014) finds that vision, hearing loss, and fine motor difficulties hamper ICT use. Explicit experiences can also be found in a usability study or in a survey that explores the attitudes of older people toward a product. Prominent theories like the (Senior) Technology Acceptance Model (Renaud and Van Biljon, 2008:216) analyze clear-cut attitudes—perceived usefulness and ease—and their influence on the adoption of technology. These models exemplify how a relationship with ICT is usually researched through visible interactions and explicit surveys. Another example of an explicit-material study is found in Álvarez-Dardet et al. (2020) who sent questionnaires to Spanish older adults to assess perceived barriers, frequency, type of use, and attitudes toward smartphones, PCs, and tablets. Our article takes a step forward analyzing implicit and structural elements.

The implicit framework delves into the unspoken and symbolic elements present in ICT use, e.g., an invisible patronizing relation between a son and the older adult that prompts the older person to disuse a technology. This framework is inspired by the field of socio-gerontechnology that critically analyzes technological solutions for older people and aging policies (Peine et al., 2021). Instead of embracing determinist notions of technology, the field appraises the entanglement of social, political, biological, and psychological factors and so forth in the interplay of technology and aging (Peine et al., 2021). An example of implicit use of technology is found in Greenhalgh et al. (2013) who encountered unexpected uses of tablets—older adults’ pragmatic customization, combining new with legacy devices—which served to study technologies against predefined yardsticks. Peine and Neven (2019:19) assert that producers of gerontechnology should not only use concepts like “acceptance” or “input” to measure the impact of technology. Instead, they should look into socio-material practices to examine race, ethnicity, gender, and other unspoken elements mediating technology practice—which this study attempts. An example of an implicit element hampering ICT use is low self-confidence, especially when first using a technology. Confidence is difficult to be reported explicitly by people, and thus, we consider it implicit. Vaportzis et al. (2017:5) found that their participants emphasized their fear of using tablets and other technology due to low confidence; confidence was the primary barrier to ICT. Horst et al. (2021) unpack confidence with technology as a psychographic feature that could reduce social isolation for older adults. They conclude that less tech-savvy participants with low self-confidence report frequent feelings of isolation.

The structural framework unpacks the political and institutional factors that go beyond older adults’ choices when using ICT. It takes a similar approach as the implicit framework since it moves beyond material interactions, but it differs in that it is large and hardly changed by an individual. For example, a structural element can involve the political management of a Senior Center, policies on aging, or the pandemic. In this regard, Twigg and Martin (2014) highlight the need to study how the “politics of age” spread to the everyday lives, and Lassen and Moreira (2014) researched how innovation policy prioritizes conceptions of aging, sidelining others. In the realm of technology and aging, López Gómez and Criado (2021) look into the politics of participatory methods among Spanish examples about telecare. Similarly, McGrath and Corrado (2019) examine the factors that trigger technology adoption for older people with age-related vision loss. Among the factors, they find institutional/political factors regarding privacy that centered around the provision of personal information when purchasing applications. Investigating other political issues in technology use, Bergschöld (2018) unfolds how nursing students learn the sociopolitical consequences of gerontechnology for older adults with dementia.

The experiences of older adults with ICT are unpacked through the concepts “barriers and facilitators” that hinder or enable technology use. Analyzing barriers and facilitators should not be understood as a way to inform how to trigger the use of ICT in the older population, since ways to support older adults are not necessarily technological. As Selwyn et al. (2003:577) say, there is a political interest in assuming that ICT is inextricably useful and desirable for older adults, which is often untrue. Rather we attempt to critically problematize the use of ICT through examining barriers and enablers. We performed the study before and during COVID-19, but these times are not central points of comparison.

Information and communication technology in this study comprises landline phones, cellphones, radio, TV, smartphones and apps, laptops, and computers. We understand ICT use as a sociotechnical practice whereby the social context is as relevant as individual use or technical features. Within the social context, we find social identities affecting ICT practice. ICT usage remains connected to social class, age, gender, social capital, and other identities. We paid special attention to the social identities of the participants because we believe they are important to technology use. Our goal is not to analyze social identities separately because social identities are present across the explicit and implicit frameworks. In the structural framework, particular stories and participants’ social identities are not disclosed because we shed light on the political structures that determine ICT use from upper positions.

Several authors draw on social identities and ICT. Cotten et al. (2016) discuss that older adults are frequently compared to other age cohorts, so they demonstrate that racial and socioeconomic variations occur within the older population to demonstrate that they are not a homogeneous group. Tan and Chan (2018:129), researching with Singaporean older adults, show that social and cultural capital are particularly central in those who are reluctant to ICT and comfortable with them. In Colombia, Mexico, and Peru, education affects ICT use more than income (Gutiérrez and Gamboa, 2010:358) and rural older adults found social and technical hurdles with ICT but gained social engagement and independence (Baker et al., 2017:16). In Spain, González et al. (2015:4) conclude that age, education, and income do not affect older people’s interest in using ICT. Otherwise, high involvement with computers prompts Spanish older adults with limited education and computer skills to view ICT positively to communicate, entertain, and stay informed.

Apart from social identities, vast quantities of literature focus on the explicit experiences of older adults with ICT, e.g., studies of usability, physical barriers, specific motivations, etc. Albeit little is known about experiences with the structural and implicit frameworks, especially among Spanish older adults.

The general aim of this research is to disclose what enables and hinders ICT practice. For that, we highlight the social identities of the participants, and we present the experiences of Spanish older people with ICT in three frameworks. In other words, this research unpacks how social identities are entangled in the implicit, explicit, and structural enablers or barriers for Spanish older adults using ICT.

## Materials and Methods

Our methodology involves three stages that are further elaborated below. As an overview, we first interviewed older adults to comprehend their experiences with tablets and mobile phones. Grasping these experiences allowed us to later intervene in a community of older adults to help them learn ICT. Consequently, we participated in and observed ICT courses for older adults, until COVID-19. The outbreak led to online participatory observation to learn how they dealt with confinement through ICT. The rationale for the different methods was to apply our knowledge in a community setting and adapt to the pandemic. Furthermore, different methods gave rise to different insights and interpretations of the data. We chose ethnography because it provides deep details about the participants’ stories. The explicit/implicit/structural frameworks are based on the fieldwork: interviews and observations gave explicit information (e.g., physical barriers and motivations), whereas participatory methods uncovered implicit feelings or structural–institutional experiences with ICT such as surveillance and management (Flow Chart 1).

**FLOW CHART 1:**
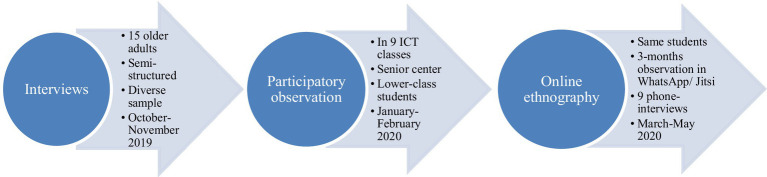
The methodology in a nutshell.

The participants come from diverse class, education, and gender backgrounds, especially in the first interviews, whereas the participants in the ICT courses came from homogenous backgrounds (rurality, illiteracy, women, etc.). A summary of the demographics, with further details below, can be found in the Table 1. After and during the interviews, courses, and online participation, the ethnographers took notes by means of explorative jottings (in a field notebook and without a predefined sheet), pictures, and screenshots. The data were later documented on shared documents for the rest of authors and translated from Spanish into English. We changed the names of the participants to protect anonymity, and the ethical committee of the Universidad Politécnica de Madrid approved this research.

**Table 1:** Summary of the demographics.

Stage	Age	Gender proportion	Social class
Initial interviews	Mean 78.5 (65–90)	10 women and 5 men	2/3 former blue collars and 1/3 white collar
Participatory observation in ICT courses	65+	Six women and three men in the elementary ICT classes; four men and three women in the advanced	Former blue collars
Online ethnography	65+	Six women and three men	Same working-class context

### First Stage: Interviews

The lead author first interviewed 15 older adults from October to November 2019. Interviews lasted 1 h 7 min on average. He took few photographs of the devices while being used and recorded audio for later transcription and analysis. For this, informed consent was given. The inclusion strategy was to interview adults older than 65, living in Madrid, with at least a basic command of ICT (they had a mobile phone and relatively understood what an ICT is). We recruited the first four older adults through an H2020 European Institute of Innovation and Technology (EIT Health) project. Then, we sought older adults in our networks (friends, relatives, and colleagues who gave us contact information for older adults). The first author eventually interviewed 11 more. Recruiting the participant through a friend or colleague enabled us to meet at the participant’s home, which helped contextualize daily ICT usage. Other interviews took place in cafeterias or hospital.

Half came from rural Spain to Madrid 60 years ago, the mean age was 78.5 (age ranged 65–90), 10 were women, and 2/3 were blue-collar workers with elementary education. We initially focused on low-income older adults, but we later kept the inclusion criteria open for several reasons. Firstly, we could not infer their income by observation, and we preferred not to ask out of politeness. Secondly, the type of job or level of education can be more representative of social class than income. A person can have low annual income, but simultaneously own properties or have savings, which better indicate social class, privileges, or vulnerability to discrimination.

The interviews were semi-structured. We initially asked the participants about their life stories and daily life: life in the neighborhood, work history, childhood, hobbies, etc. This information enabled us to understand, for example, how being a woman/man and blue−/white-collar worker affected their lives. Then, shifted the discussion to smartphones and tablets as these ICT were the focus by the time. We discussed general and applications use, motivations, barriers, learning process, feelings, and emotions about ICT (see interview guide in Supplementary Material 1). As the interviews progressed, the participants included other technologies (radio, TV, computers, and landline phones) when talking about phones/tablets, so we broadened the scope.

### Second Stage: Participatory Observation in ICT Courses

The insights generated from the previous interviews acquainted us with the interactions of older adults with ICT, however reporting experiences with technologies verbally without observation stayed insufficient. Consequently, the lead author approached a different group of older adults at a senior center. In Madrid, there are several public senior centers where different activities (computers, yoga, games against memory decline, etc.) take place for dwellers over 65 years old. These centers are financially and logistically supported by the City Council. The courses are organized routinely, and the older adults go through an enrolment process and must attend them if accepted. The activities are consistent with the active aging policies to support the health and prevent the loss of autonomy of older adults. He observed and participated in two ICT courses from January to February 2020, since we were not allowed to have more than one attendee. In the courses, the ethnographer observed the students dealing with computers and smartphones: emotions, complaints, motivations, physical struggles, teaching style, the center’s management, and ICT affordances. Later, aside from observing, he helped students carry out their tasks simultaneously. Helping the students allowed the ethnographer to build rapport with them. The students started to be more open and to share their life’s stories (which strongly intersect with ICT use as this article unfolds). This was eventually helpful to grasp their barriers with and enablers of ICT.

We attended nine classes in total at the senior center: six focused on computers and three on smartphones. Four classes were for elementary students of ICT and five for advanced. Each level had a different teacher, and there were approximately nine attendants—mostly women in elementary ICT and men in advanced. Their age was 65+. The tasks were emailing, use YouTube, Drive, and Word, and each lesson lasted 1 h 30 min. The classroom had 11 old computers—yet working well. The main problem rested in the inoperative Wi-Fi connection provided by the municipality, which is later analyzed as a barrier for the students.

The senior center is in the neighborhood “Orcasitas,” situated within the 31 percent poorest areas in Spain and 10 percent in Madrid (INE, 2018b), and where the students live. Through talks with the center’s manager, we came to know about the overall students’ lower-class background: the majority of them have low level of education, rural origins, they live in social housing, and are known for being politically active (during the fieldwork, the students participated in demonstrations asking the authorities to remove asbestos from their blocks). This group differs from the first older adults interviewed who had more diverse backgrounds. Diversity of people enabled us to compare the experiences with ICT from different angles, e.g., a woman who worked as a seamstress or as a housewife probably lived different barriers and enablers with technologies than a white-collar man. Furthermore, broadening the inclusion criteria allowed us to not focus on a particular group whose social identities are difficult to infer.

### Third Stage: Online Ethnography and Phone Interviews During COVID-19

When COVID-19 grew, between March and May 2020, the lead author kept in touch with the same students of the ICT courses and the teacher *via* WhatsApp groups and video chats on Jitsi. We chatted, exchanged videos and pictures, and solved riddles and games. These interactions were captured to observe the limitations and positive influences of ICT, how WhatsApp and Jitsi interfered, how teaching ICT evolved and could be otherwise, and skepticism toward the management of the pandemic through using social networks or TV. The pandemic itself was not an analytical category, but how COVID-19 transformed the ways we experienced technologies and how participants expressed emotions/feelings through ICT.

WhatsApp and Jitsi enabled us to overcome the COVID-19 challenge and to research ICT practice by getting involved and observing online on the platforms. However, for the students who did not know the ethnographer, online research hindered an empathetic relationship (unlike in-person interactions). We also lacked insights from participants who were members of the WhatsApp groups but were not active.

We also phone-interviewed seven students of the advanced class, one elementary student, and one from our networks. The ethnographer contacted them on WhatsApp to arrange the interview and the advanced students participated more in the chat, which explains the gap between the advanced and elementary students interviewed. With the interviews, we aimed to unpack the role of ICT during the beginning of COVID-19 through oral means that we lacked on WhatsApp and Jitsi. In particular, we discussed barriers with and enablers of ICT, how they kept in touch with loved ones and the doctor, apps use, change in perception of ICT, and emotions and feelings on the pandemic expressed through technologies (see Supplementary Material 1.1). The interviews lasted around 20 min, and we transcribed relevant fragments.

### Data Analysis

We initially wanted to unfold the dichotomy barriers/facilitators in ICT use. The data generated in the fieldwork pictures, transcriptions, and field notes were first transcribed, coded, and illustrated in nodes on NVivo by the lead author. NVivo is a software that enables a researcher to analyze and code data, mostly qualitative. Even though the data came from different methods and participants, the data were grouped in nodes transversal to the different sources, i.e., we triangulated the data. The triangulation of methods consists of using and converging data from different participants and settings (Patton, 1999:1189), and it enabled us to capture different dimensions of the experiences with ICT (e.g., before and during COVID-19). The other authors reviewed and discussed the codes. The resulting and main nodes were barriers and facilitators grouping subthemes (physical barriers, motivations, affordances, usability flaws, etc.). However, these elements stayed short, and we later aimed to analyze beyond explicit patterns (visible or spoken barriers/facilitators). Therefore, we used situational analysis of Clarke and Friese (2007) to reinterpret the nodes of NVivo.

Situational Analysis is a methodology that uses situational maps to analyze a particular situation by identifying and representing the complexity and variation of data, reveal marginalized perspectives, and empirically decenter the “knowing subject.” Situational maps intend to display major elements of situations (e.g., discourses, humans and non-humans, controversies, collective elements, symbolic/sociocultural, political/economic, temporal, and spatial factors), and incite analysis of intersections and divergences among them (*Ibid*.). These maps are suggested along with Social Worlds and Positional Maps, but we only used situational maps because they were sufficient to uncover what elements are at stake in the interactions of older adults with ICT. The maps are not intended to illustrate “findings” or truths about a circumstance. Rather, maps are to be viewed as an interpretation of a situation that is constantly changing.

By mapping major elements in ICT practice, we identified different technologies used; participants (teachers and older adults); affordances; facilitators (e.g., motivations); physical and silent barriers (e.g., cognitive decline and surveillance); institutions; the pandemic; lack of facilities in homes and the classroom; contradictory uses of ICT; social identities (e.g., gender roles); and attitudes toward ICT (e.g., adversity). The latter portrayed a messy map that can be seen in Supplementary Material 2. Then, the lead author connected the elements by colored lines to unwrap intersections and divergences. The rest of authors reviewed the connections, and we perceived that social identities were not categories *per se*, but they cut across other categories.

We then organized the elements in an ordered template retrieved from Clarke et al. (2018) that can be found in the next table. The template triggered us to separate the individual and collective human actors; non-human elements; silent actants; discourses upon non-humans; debates; and symbolic, political, temporal, and spatial elements. Finally, splitting up the elements in the ordered table allowed us to identify three overarching analytical frameworks hindering or facilitating ICT use. The first deals with implicit-silent elements (e.g., unwritten rules, gender roles, low self-esteem, and contradictions). The second encompasses explicit-material experiences (e.g., physical limitations, ICT affordances, clear preferences, etc.). The third spans political and economic structures (e.g., lack of facilities, COVID-19, and economic limitations). These frameworks serve to analyze the experiences of older adults with ICT from visible, unspoken, and institutional frameworks that form the following section (Table 2).

**Table 2:** Ordered version of elements that constitute this research. Template retrieved from Clarke et al. (2018).

**Individual human elements/Actors**	**Non-human elements actors/Actants**
Fifteen initial and diverse seniors; seniors in the senior center; teachers; seniors during COVID-19; researcher (participant, observer, and interviewer); seniors’ children and grandchildren; and wife–husband.	Cellphones; smartphones; tablets; computers; laptops; landline phones; WhatsApp; TV; radio; Jitsi; Facebook; camera; transport app; health apps; and newspapers and other apps.
**Collective human elements/Actors**	**Implicated/Silent actors/Actants**
Senior center; government; and ICT stores.	Fear; insecurity; carelessness; loneliness; patronizing relation son-older adult; contradictions speech-performance; unwritten rules; teaching system; and discriminating identities.
**Discursive construction of non-human actants**	**Major issues/debates**
Barriers (usability, design standards, physical—hand tremor, finger clubbing, low vision, and frailty—and mental barriers—memory, speed); surveillance; information retrieval; adversity; associated practices; motivations (safety, communication, entertainment, and independence); big or absent interest in ICT.	Are we leaving out identities of older adults and are we pre-assuming some identities? Where were the identities more emphasized? Is a socio-political approach actually relevant to ICT usage? Does ICT facilitate or hinder seniors’ lives? Should design be accessible or should we train older adults to use normalized standards? Why is the State concerned about older adults’ learning ICT?
**Sociocultural/Symbolic elements**	**Political/Economic elements**
Social capital; social class; political activity; rural background; elementary or higher education or cultural capital; absent or large work experience with ICT; gender differences; income; cosmopolitanism; and privileged vs. non-privileged; all seniors living at home.	Lack of facilities in the senior center and in seniors’ homes; economic constraints of participants; and social and political history of neighborhood.
**Temporal elements**	**Spatial elements**
Before and during COVID-19	Spanish, Madrid, and Orcasitas context.

## Analysis: Experiences of Spanish Older Adults With ICT

*An older woman does not understand WhatsApp. She thinks every group’s message is for her and, regardless of the content, answers with the same voice-recording: “María, I saw your message, I’m alone at home waiting for my son to come, bye. She occasionally shares voice-recordings sobbing and claiming she’s lonely. In the course, she was very low skilled in ICT. […] She misses her late husband* (Ethnographer’s field note of WhatsApp during COVID-19).

We met her in the elementary ICT course, and, during COVID-19, she showed perceptible barriers typing (low vision and hand tremors), so she used voice recordings instead. She seemed not to grasp some unwritten rules of WhatsApp: no need to reply to every message. Her remaining obstacles were gendered and educational: illiteracy, widowhood, depression, loneliness, and inexperience in ICT (her husband used to handle ICT). She represents an example of embodying ICT through an intersection of explicit, implicit, and structural barriers. The following analysis is divided into three sections; however, some examples like this one might better be discussed through an intersection of explicit, implicit, and structural frameworks (this is reflected in the discussion section). The next table is shown to visualize in a nutshell how the different experiences of the participants are organized (Table 3).

**Table 3:** Summary of the dimensions.

Implicit-symbolic situations	Surveillance and paternalism hampering ICT use: older adult children and their parents. Feelings and emotions: fear of improvisation, low self-esteem, etc. Contradictory speech and actual use of technology.
Explicit-material interactions	Possession of devices Physical limitations hindering ICT use: limited memory, low vision, etc. Clear-cut motivations facilitating ICT practice: follow your team, entertainment, health. The role of WhatsApp and Jitsi in COVID. Phone’s affordances: software, hardware, and smartphone models.
Structural-institutional barriers	Political design (lack of facilities) and teaching at the senior center. Political skepticism in COVID.

### Implicit Situations

This type of interactions involves symbols or unspoken representations observed by the researchers that facilitate or hamper technology use. We generated these interpretations upon situations, e.g., when the participant used a computer in a class or a comment given by the participant (the words did not matter *per se*, but the representation of the comment such as an emotion). Social identities, such as lack of education in ICT, gender, or type of social class, are represented in the invisible experiences with ICT.

#### Implicit Situations: Surveillance and Paternalism

Participants often mentioned their children in the interviews in a way that hindered their use of ICT. We observed a strong patronizing relationship between the adult child and older adult. Adult children taught them how to use ICT, bought the devices, checked the bills, supervised them, etc. For example, most of the initial participants used the former relatives’ mobile phones, and nine older adults stated they were taught ICT use by relatives. Carmen depicted how her son supervises her using ICT: *My son tells me not to pick up the phone, except to call. […] Everything is set up by my son […] My son orders me: the tablet is to play* (Carmen, widow).

Numerous adult children required their parents to carry the cellphone outside for safety. But several participants did not take the cellphone outside in order not to be monitored by their children. As such, ICT disrupts Ana’s life: *I do not take the phone outside, otherwise, they know where I am. […] When I am home, they never call me, but when I go out, they call me* (Ana, widow).

Other older women portrayed surveillance differently. Josefa (with vast work experience in ICT) refuses the geo-localization features, which stems from her industry experience. Nuria is also an ICT expert and deemed geo-localization as an inherent hazard: *It’s like a toll to pay when using Google. At my age, I benefit more than I risk* (Nuria). While for the first women without much ICT experience, social bonds influence her relationship with ICT, no relatives mediate Josefa’s and Nuria’s usage.

#### Implicit Situations: Feelings and Emotions Toward ICT

Many of the initial participants rejected ICT for replacing face-to-face communication, developing addiction, etc. Others felt it is impossible to keep track of its rapid evolvement acting as a barrier. Four older adults, despite using ICT regularly, despised it. Throughout COVID-19, the students with prior interest in technologies regarded ICT positively. Confinement was a facilitator because it triggered technology use for some, whereas others kept using ICT as before. A male student defined ICT to tackle isolation: *If it wasn’t because of ICT, I would not talk with my sons. (COVID-19) would be way worse without ICT*. (Francisco)

In contrast to the positive views, low self-confidence arose among the older adults inexperienced in ICT which hindered their use. The participants who kept their cellphone over a new smartphone lacked the self-confidence to learn the latter. They associated this decision with age and felt comfortable with cellphones for simplicity. One linked simplicity to low self-esteem: *I asked him for a phone for rednecks, to call and receive calls* (Alberto, man elder).

We observed the students (from the lower class and with elementary education) following the indications from the teacher cautiously. In this sense, they revealed a fear of improvisation and self-doubt, which can stem from the old school system. Moreover, they often deleted the whole text when mistyping something, instead of placing the cursor at the wrong character possibly based on a lack of self-confidence.

Linking fear of economic fraud and lack of experience in ICT, an older woman expressed reluctance: *as the cellphone entails so much fraud, I do not want to get into trouble* (Laura). Additionally, one person dreaded testing apps during COVID-19: *I am scared of getting into a place and busting something or being charged* (Francisco).

#### Implicit Situations: Contradictory Speech-Act

Although several older adults had a negative attitude toward ICT, we experienced inconsistencies associated with low self-esteem that remain common among women and lower-class participants. They did not know the differences between cellphones and smartphones, but later, they demonstrated awareness. Others ignored what a smartphone was, despite possessing one. It seemed they expressed blindness toward ICT, regardless of competent performance and frequent use:

*If I ask them a direct question on ICT use, they ignore it or disuse it. But after follow-up questions or observations, they know what I mean! They are quick to say NO! It may be due to low self-confidence* (Field note of an interview).

### Explicit Interactions

This type of experiences entails material situations that were either easily visible by the researcher or were explicitly commented by the participant. For example, a flaw in the design of a product or the hands of a participant is clear things that hampered ICT practice. We perceived other situations as facilitators. These interactions were associated with different social identities. To begin with, the older adults possessed different devices:

Of the initial participants, they all possessed a mobile phone, except two. Half had smartphones and used the phone at least once a day, and five had a tablet. In the computer courses, almost all students possessed a smartphone, regardless of low experience with ICT, illiteracy, lower social class, etc., because of prior interest in ICT and relatively young age (65–70). Few advanced students owned a laptop or a desktop, and those were the only ones having Wi-Fi access at home. All the participants approached during COVID-19 had a smartphone, 3/9 had a tablet, and 5/9 had a computer.

#### Explicit Interactions: Physical Limitations

Ten initial participants faced severe bodily hurdles. Limited memory hindered recall of the steps to reach apps and phone numbers, and numerous students could not remember their email passwords. One saved the password written on a paper and, when typing the password in the box, she wrote “password + actual password,” which showcased significant literacy limitations. Another male older adult—sobbing—complained about his limited memory caused by a stroke: *I get blank dullness and sometimes I cannot solve issues […] I remember her phone number, but not my sons’* (Juan).

Several participants experienced poor thumb performance due to finger clubbing and hand tremors. They could not click accurately on the touchscreen or keyboard. They used to perform manual labor, symbolizing a convergence of lower social class and poor health. This condition limited an older woman, who all her life worked as a seamstress. She faced triple discrimination related to social class, gender, and age: *her fingers are thicker than mine since she worked as a seamstress which has affected her hands. That makes her struggle with typing* (Field note at the computer courses). Furthermore, many students could not click twice on the mouse and keep it still because of hand tremors, so they could not progress. It was also confusing for them that sometimes one click and sometimes two are needed, depending on the app, folder, etc.

Many others struggled with low-vision such as myopia and cataracts: they could not find things on the phone screen that forced them to wear glasses. Other limitations included deafness that hindered hearing calls.

#### Explicit Interactions: Clear-Cut Motivations to Use Smartphones, Tablets, and Landlines

Although Ana rejected ICT, she tracked the updates of the Real Madrid club through her smartphone. Carmen used the smartphone to visit her husband in his nursing home. Despite their gender barriers, elementary education, and lower class, they actively used smartphones and other ICT. Other reasons to use smartphones included communication with relatives and health tracking through the official online e-appointment mobile application for primary and specialized care (i.e., “Cita Sanitaria Madrid”), which proved useful to delay appointments during COVID-19.

Smartphones were deemed beautiful and useful, which proved as a facilitator. The owners of smartphones rejected cellphones, which makes us consider that smartphones—regardless of usability problems—prove more helpful than cellphones: *I’m keener on smartphones than the classics, it’s another style, not that brick, it’s more useful, for example, you can keep it in the pocket. I stare at phone stores to see how beautiful they are* (Ana).

Tablets were mentioned mostly among the initial participants. Elena and her partner used the tablet to check recipes, fashion, transport, trips, and forecast. Her partner was not fond of the tablet but preferred the computer. The latter shows a gender disparity: He defined the computer as more sophisticated and he handles the sophisticated devices at home. This can hinder Elena from adopting ICT. Another participant frequently used her tablet for entertainment board games, online newspapers, etc. She connected a political stance to her tablet: *How can I get rid of the kings? –playing solitaire on the tablet– […] I cannot bear the kings laughing* (Carmen). Carmen preferred a tablet over a smartphone for its size and entertainment apps. During COVID-19, Begoña enjoyed the increased free time that enabled her to spend more time with the tablet—the pandemic acting in this way as a facilitator.

Seven of the initial older adults opted for landlines over mobile phones. They considered landlines easier and with a longer shelf life. They better remembered landline numbers, and two participants remarked on the landline’s cheaper flat rate. These features enabled ICT practice. Landline users overall rejected ICT, as they did not feel familiar with the technologies, and this was highly marked by social identities. These participants did not possess ICT experience, higher education, nor did they belong to the upper-class.

#### Explicit Interactions: WhatsApp and Jitsi Interceding During COVID-19

All elementary and advanced students felt familiar with WhatsApp. However, its design hampered the use of the app. The elementary students communicated by voice messages since they struggled with typing due to small keyboards and illiteracy. During COVID-19, we shared games, riddles, jokes, and computer tasks, and talked about how we were doing. The advanced students participated more on WhatsApp than the elementary group since they handled ICT better.

WhatsApp could act as a facilitator. The students employed WhatsApp video calls to keep in touch with relatives and friends. Luisa reflected on a positive outcome of the outbreak and WhatsApp: *My neighbors and I communicate more on WhatsApp. We are more united for coronavirus. Before, we did not see each other in a week* (Luisa, advanced student).

**Figure 1:**
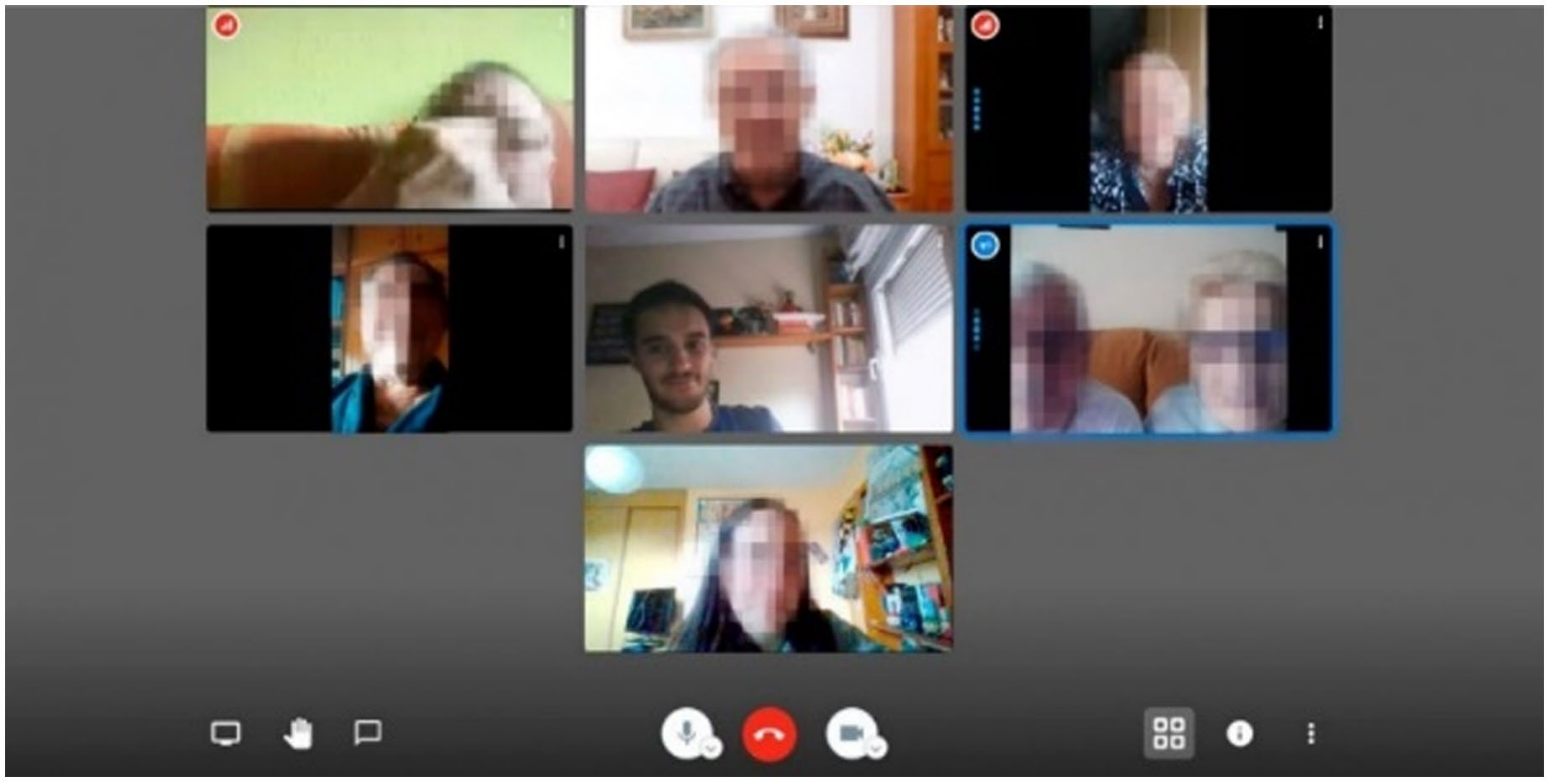
One of the talks on Jitsi with advanced students of information and communication technology (ICT).

The students, the teacher, and the ethnographer continued meeting during confinement on Jitsi, a videoconferencing app. The app proved problematic for the elementary students since only one participated. The latter might derive from a fear of trying new things. In contrast, the advanced students seemed eager to employ Jitsi, two even asked the ethnographer to set up a call for their families. Many students deemed Jitsi as the only new app learned in confinement, i.e., COVID-19 did not heavily promote the use of new apps for at least nine older adults (Figure 1).

Jitsi posed barriers for a few students. The ethnographer shared the call’s link on WhatsApp, and since many did not own computers, they logged through smartphones. On smartphones, they needed to download the Jitsi app and a few students struggled with the information displayed in English when downloading the app. The owners of computers initially could not log in because they mistyped the link without an accent. Furthermore, none had WhatsApp installed on their computer to open the link directly. With help most of the students completed the process, except for a couple. During the call, they were unfamiliar with videoconferencing, so conversations often overlapped. These practices embody cultural and economic barriers in ICT: lack of Spanish instructions (Jitsi designers expect everybody to understand what “download” means), computers, and experience.

#### Explicit Interactions: Affordances of Mobile Phones

This section examines specific software, hardware, and smartphone models. The older adults with smartphones were eager to use the camera. For example, Félix and Natalia—reluctant users of ICT—had a smartphone only to take pictures. Pedro and Elena appreciated photographing their granddaughter and, once, provided proof of their leaky roof to the insurance company. Most of the students handled the camera well, but a few elementary students did not know how to take videos.

A male advanced student mastered his iPhone 7 and was facilitated by its font large enough and easy to read. Regarding barriers, Ana had low vision and disregarded adjusting the font in the sports app Marca, only using the teams’ logo for guidance. She could not handle the keyboard in WhatsApp either, so she used voice messages. A Sony user complained about its small letter size. We got confused when adjusting the letter size since the phone calls it “font-size” instead of “letter size,” a counterintuitive translation in Spanish. The edge of the Sony touchscreen barely worked which made the older person disuse certain letters.

In the elementary course, many students could not send voice messages and pictures on WhatsApp, but were enthusiastic when the ethnographer taught them. The students also got confused with the two microphones appearing together on WhatsApp—one to convert audio to text and another to send voice messages—which proved problematic for those struggling with hand tremors or finger clubbing.

The advanced students managed their smartphone well but often pressed the start button because of the small touchscreen and hand limitations, which, for example, made them lose track of emails being written. More barriers were found in another student had an LG with information displayed in English, despite the system’s setup being in Spanish. Other students only had access to an online gallery that required an internet connection, which only a few had. Carmen experienced several challenges with her Sony XA1 Ultra and grumbled about not getting notified when the billing period ended or she neglected to hang up.

Regarding cellphones, one-third of the initial participants had an Alcatel 2008G. They considered it easy to unlock, mute, place, and take a call, and dial numbers (for those with proper vision). It costs around 40 euros and Spanish phone stores recommend the cellphone as senior-friendly. However, its owners generally did not master ICT, had elementary education, and belonged to the lower class. Its letters are too small, and three letters appear on each number of the keyboard, which hinders texting. The problem does not only lie in usability, but in why the cellphone is marketed as senior-friendly. Older adults may ultimately reject other ICT because they do not feel Alcatel 2008G helps their lives.

### Structural Barriers

We consider that structural barriers stem from the institutional or societal level and are hardly transformed by an individual. For instance, the management of the senior center and its decisions on the design of the ICT classes, or the pandemic, are factors that influence an older adult using a technology. Here we did not observe structural facilitators, apart from the opportunity to learn ICT for the older dwellers. Except for two examples, the point of this section is not to unfold stories of particular participants or their social identities because our goal is to shed light on how the practice of ICT is arranged politically. This framework is rooted in the highly specific context of the senior center (where the fieldwork was held), so the generalization of the reflections is difficult to be applied somewhere else.

#### Structural Barriers: Political Design and Teaching ICT at the Senior Center

The center, where computer courses took place, had flawed and exclusive facilities. In the advanced course, a disabled woman could not reach the mouse nor keyboard from her wheelchair. She required her husband’s help and got lost following the teacher’s assignments. She did not switch to the elementary course because she wanted to remain beside her husband who preferred the advanced course. The ICT classroom thus excluded people with functional diversity. Not adapting the facilities depends on the municipal government, and it has likely prevented many older people from learning ICT.

When the center’s Wi-Fi did not work, a tiny “*x*” appeared on the top of the Wi-Fi symbol and was hardly noticeable. The students could not connect to the internet, and they often ignored the source of the problem. The Wi-Fi proved to have other shortcomings:

*It is really tough to reach the public municipality’s Wi-Fi. Every 2 weeks the student needs to log in again typing their names, email (whose accounts do not remember), phone numbers*, etc. *The system messages you a different password each time. The students must go back and forth to the messages inbox, copy/paste the password, go to the website… being unnecessary* (Field note of the senior center).

The decision to modify Wi-Fi relies upon the political power that, for the time being and neglects senior-friendly design. Thereby, the municipal government does not seem to invest much effort in centers for older adults.

Another political issue stems from a transit app to know when buses arrive. In the courses, the students were eager to learn how the *EMT Madrid* app works so as not to wait buses unnecessarily. The political controversy lies in that they are forced to use the app because there are no metro stations near the students’ homes, lacking access to other parts of the region.

The method of ICT instruction proved inequalities. During COVID-19, the main teacher shared Word and Excel assignments on WhatsApp. Only a few of the advanced students completed them since the rest did not own computers nor Wi-Fi access at home. We thereby questioned the relevance of computer lessons:

*The students started by writing emails, but do older adults around 75 need an email account? If they want to buy online stuff, ok, but not sure about other things. […] A student was more interested in learning smartphones than computers as she does not have one at home […] They say phones are easier to handle than computers* (Field notes of the senior center).

Lessons on smartphones could engage students better. Computers often cost more than smartphones and require genuine interest and experience in ICT. The problem lies in students’ economic limitations and lack of ICT experience. Nevertheless, changing the course relies upon the managers. Hence, it is political, and it seems the managers did not consider the realities of older adults.

#### Structural Barriers: Political Skepticism in COVID-19

Information source retrieval affected the relationships between ICT and older adults during the pandemic. COVID-19 affected us all in a manner that we could not control and in this way it became structural. Some participants perceived WhatsApp and Facebook as fake news spreaders, so they used other means: *I prefer to check if it’s real in the newspaper. Most of it –on WhatsApp– is fake news* (Teresa during COVID-19). Others preferred TV or radio to retrieve information, since these tools are switched on all day at home, except for a few who grumbled: *TV lies to us*, e.g.*, Severo Ochoa. […] They show what they want us to know. There is so much coronavirus information […] It overwhelms me* (Teresa).

COVID-19 polarized the political situation in Spain. This scenario arose on a Jitsi videoconference when an alt-right supporter argued with other students about the necessity of criticizing the government during the crisis. In this sense, the pandemic became political and hampered the older adults’ trust in technologies to get informed and communicate.

## Conclusion

This study contributes to a field wherein analysis of material-explicit experiences of older adults with ICT prevails: usability tests, surveys of attitudes with ICT, and predicting models of technology adoption such as “Senior Technology Acceptance and Adoption Model” (Renaud and Van Biljon, 2008:216), etc. Acknowledging the usefulness of these for policy-making and technology design, this article goes beyond by uncovering how social identities enable or hinder ICT practice through implicit, explicit, and structural experiences. First, we introduce a summary of the results related to other studies. This is followed by a theoretical discussion.

Implicit situations included barriers and opportunities. Children surveilling older adults through smartphones, and inconsistent ICT use and speech hindered their relations with technologies. The last to our view derives from misperceiving older adults as inoperative with ICT: younger people do not expect older adults to handle technologies, so older adults embrace this social norm and reject ICT. Low self-esteem and fear determined ICT usage among females, people inexperienced with technologies, and lower-class older adults, so we suggest that ICT training can empower unprivileged groups, as Ratzenböck (2017:25) suggests for older women. Czaja et al. (2006) point out that self-confidence decreases as we age, which is roughly corroborated by our research. Vaportzis et al. (2017) point in the same direction. On top of this, we add that low self-confidence is higher among those not tech-savvy or with unprivileged identities. We found opportunities among the students of the center who viewed computers and smartphones positively. The students had motivations to attend computer courses, and their unprivileged background did not affect their interest in technologies, which resonates with González et al. (2015:4). Therefore, motivation, education, and social capital can be better enablers than income in ICT practice, which Tan and Chan (2018:129) and Gutiérrez and Gamboa (2010:358) also argue.

Regarding the explicit interactions, physical barriers included limited vision, memory, auditory, and thumb performance, which is in line with previous research on barriers to the use of ICT (Gitlow, 2014). Privileged participants (upper-class and men) had tablets and computers. They used smartphones to track health and communicate, whereas tablets were employed for entertainment. Having privileged identities portrayed strong facilitators of technology use. Cotten et al. (2016) also unpack privileges when it comes to using ICT; they argue that among the older population there are striking differences in the Detroit area between those with higher income and white compared to African Americans and poor. However, the assumption that an unprivileged background leads to disuse of ICT is wrong, at least among these participants. We find that motivation or belonging to a strong social network might better facilitate ICT use than social class or gender. Lower-class participants without experience in ICT embraced landlines—which differ from Petrovčič et al. (2016:100) who found gender to be a better predictor than class. In this way, social identities play a big role when triggering or hindering ICT use. WhatsApp and Jitsi helped the students get over confinement, and facilitated technology use, but posed cultural and economic barriers for those lacking computers. The participants appreciated smartphones over cellphones, though complained about smartphones’ small interfaces and keyboards. Older adults, therefore, do not need out-of-date devices to adopt ICT but training.

**Figure 2:**
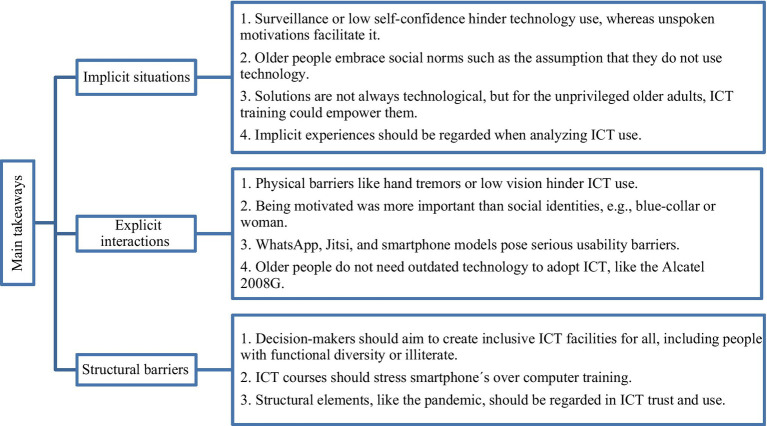
Main implications of the study.

Regarding structural barriers, it should be noted that the barriers are very specific to the context of this study in Orcasitas (Madrid). The political or institutional agents most likely work differently in other places. We found that reaching an inclusive design of facilities and ICT for older adults concern the decision makers of the senior center and governments. In the center, we encountered exclusive conditions for people with functional and class diversity: Wi-Fi, transport, and classroom design. The instruction method disregarded older adults’ desires since it did not include smartphone training. These elements hindered ICT practitioners living in the Spanish neighborhood “Orcasitas” from learning ICT and, thereby, we struggle to find facilitators. We wonder whether these conditions existed in a senior center located in a wealthier area. Moreover, authorities should also consider that COVID-19 polarized older adults politically and structured how they trust technologies. An example can be found in Rudnik et al. (2020) who discuss the experiences of the “oldest old” older adults engaging and sharing political issues through ICT. Technology represents an opportunity for increased engagement in politics but offers drawbacks such as overwhelming information or being left out of politics if the older person is not tech-savvy. Rudnik et al. (2020) acknowledge that political participation is frequently unrecognized in research. Similarly, our participants debated the responsibility of the government for the pandemic in a video call, meaning that structural issues surround the manner older adults learn and practice ICT. As López Gómez and Criado (2021) and Bergschöld (2018) do, we draw attention to the political arena when it comes to studying technology for older adults. A summary of the findings can be seen in Figure 2.

### Theoretical and Analytical Discussion

After the summary of the results, we discuss how our theoretical and methodological approaches could be compared with others. Concerning social identities, our premise is that older adults comprise complex and intersecting identities that influence ICT use, i.e., they are not simply lower class or old. Conceiving of identities as intersectional helps comprehend ICT practice not only as a matter of age but related to gender, education, etc. For this, the notion of intersectionality is helpful. Crenshaw (1991) introduced the notion to analyze the intersections of social identities and their discriminations. The theory posits that identities are fluid and shaped by structures and social processes, and people cannot be reduced to single categories, nor can single categories depict understandings of individuals (Hankivsky, 2011). The notion could enable us to analyze how age, race, class, gender, and other social identity markers intersect depending on how these identity markers eventually discriminate or privilege older adults’ use of ICT. However, this study shows that being unprivileged can be deceiving for somebody who is otherwise privileged. We opened our inclusion strategy with participants: we could not infer discriminations by examining their incomes, as they might be privileged through, e.g., property ownership. Moreover, qualitatively analyzing which identities reign over others in ICT usage proved blurry, e.g., uncertainty whether gender or class prevails. We did not ask the participants how they fit into gender, class, etc., out of politeness, so the identities are depicted based on observation, which might have led to misrepresentation.

Star (1990:47) argues that dichotomies (e.g., privileged–unprivileged) are exclusionary: marginalized groups are not simply left out, but they are in a “high tension zone” aiming to be inside. Star (1990:26) examines the role of technology standards in fostering marginality. For her, non-marginalized networks create standards in technologies that deny multiplicity and contingency in favor of stability and unity, and dismiss how standards could be “otherwise.” The seamstress woman or the students struggling with the Wi-Fi password represent how ICT disregards standards for people with gendered, literate, class, and physical disadvantages. Star (1990:45), therefore, sheds light on the “high-tension zone” to unpack the properties of conventional and standardized networks, which this article attempts to reveal, e.g., with the political networks at the senior center.

Although we cannot generalize, the ethnographic approach enabled us to uncover unspoken experiences from different angles and provided us with reflections that other researchers may encounter. The implicit, explicit, and structural frameworks emerge from the situational analysis performed, for that; we encourage researchers to analyze situations as Clarke and Friese (2007) suggest to avoid the “analytical paralysis.”

The implicit and structural frameworks could be associated with López Gómez (2014) notion of socio-material arrangements that older adults use to appropriate technologies. Socio-technical arrangements are mundane and material things that we do to keep our lives in order. Older people use technologies through these preexisting and little arrangements in their daily lives, and these are not often regarded by the gerontechnology industry. The notion is inspired in Actor-Network Theory which posits that observations should emerge from the voice of the researched participants and not from conceptions that researchers pre-assume. We employ implicit and structural markers and the industry does not commonly consider them either. However, implicit/structural differs from *arrangements* in that ours are observations by the researchers, and hence, they do not emerge explicitly from the participants. López’s approach could be better associated with the explicit framework of this article, since that framework attempts to represent more directly the participants’ stories.

### Limitations

The structural, explicit, and implicit frameworks are formed upon the elements that surround and constitute the field. These might not work in different contexts but might serve as inspiration. The frameworks may resemble the triadic reciprocality (Wagner et al., 2010:871) and the “Senior Technology Acceptance and Adoption Model” (Renaud and Van Biljon, 2008:216), but these only delve into material interactions. However, some elements may have been left out by our conceptions of implicit, explicit, and structural. We have divided the analysis into three sections, but these could probably be represented into one. Certainly, the participants’ stories are simultaneously embedded into, for example, political conflicts, patronizing relationships with their children, and problems/facilitators with the design of their mobile phone. The older woman that follows the beginning of the analysis section is an example. However, splitting them up helps untangle ICT use in different realms. These dimensions need to be considered and we assert that the understanding of older adults’ uses of technology does not finish here, and more need to be studied.

Concerning the dichotomy barriers/facilitators, we found more barriers than facilitators in technology use, given that older adults often struggle more than youngsters with technologies due to design flaws and other invisible/political elements. Some may argue that an experience with technology is not necessarily unpacked through barriers-facilitators. An experience can be expressed, for example, through an emotion, a story, or an encounter. Being this true, the dichotomy helped us narrow down the experiences into tangible issues rather than into fuzzy concepts. This eventually allows the understanding of ICT practice for a broad range of fields like design, gerontology, research, etc.

The sample size was not large and should not be believed as a full representation of the older Spanish population. In a different scenario with wealthier older adults, the research could have led to different results: a better adoption of ICT, more perceived usefulness, etc. Our participants with previous white-collar jobs at least pointed in that direction. If the seamstress woman (with limitations in her hands) had had a desktop job, now she could have higher dexterity with touchscreens. Nonetheless, the sample size allowed us to dive deeply (ethnographically) to discuss the implications and uses of ICT in a segment of older people that can share social class, gender, and other experiences with other older adults. Ethnography, as a method that collects data from people, entails a certain bias, and this possibly limited our research findings. The authors do not live the daily experiences of an old person and that could affect the results. However, we could take distance from the research without becoming too involved, and all authors reviewed the collection and interpretation of the data to reduce possible bias. More possible limitations are that this investigation lacked caregivers, older adults’ children, older adults living in nursing homes or in a frail condition, and senior centers in wealthier areas. Future research could include these populations to get different and meaningful experiences with ICT.

### Implications

This study gives voice to underrepresented older adults and enables engineers and designers to understand the complexity of older people. When designing technologies, they should consider that social identities, symbolic, political, and economic elements mediate ICT practice. Moreover, designers should not stigmatize older people by marketing out-of-date products for them (e.g., Alcatel 2008G or the similar Jitterbug Flip) since these discourage older people to use current technologies. Even though this study focuses on ICT, engineers should understand that solutions to improve older adults’ lives are not always technological, as Selwyn et al. (2003:577) comment. Similarly, stakeholders should not assume that the only ways to improve older adults’ lives are only related to health monitoring or healthcare. These actions are often based on ageist assumptions, so we suggest stakeholders previously study older adults’ needs and co-create things with them to improve their lives.

Apart from considering symbolic and political elements that affect privileged and unprivileged older people, Spanish policymakers should pay attention to material issues. In particular, they need to enhance ICT facilities at public centers for older adults and their homes. These, alongside nursing homes, remained jeopardized by COVID-19, so the administration should strengthen supervision. They should also understand that tablet and smartphone training can help mitigate isolation. Finally, society (including older adults) must avoid ageist norms with ICT, such as thinking that older people are unable to use technologies, which are often unreal and older adults embrace as their own. The stereotype eventually prompts older people to disuse technology.

## Data Availability Statement

The raw data supporting the conclusions of this article will be made available by the authors, without undue reservation.

## Ethics Statement

The studies involving human participants were reviewed and approved by Universidad Politécnica de Madrid, as this study belongs to the project “POSITIVE: Maintaining and improving the intrinsic capacity involving primary care and caregivers.” The patients/participants provided their written informed consent to participate in this study. Written informed consent was obtained from the individual(s) for the publication of any potentially identifiable images or data included in this article.

## Author Contributions

XF and EV-M presented the idea of the research. MG-H and SA developed the theory, data analysis method, and designed the investigation. MG-H performed the methods and made the final manuscript. SA, XF, and EV-M supervised the fieldwork, findings and the final manuscript. All authors contributed to the article and approved the submitted version.

## Funding

This work is supported by the European Project POSITIVE (Maintaining and Improving the Intrinsic Capacity Involving Primary Care and Caregivers; reference 20683) funded by the European Institute of Innovation & Technology Health.

## Conflict of Interest

The authors declare that the research was conducted in the absence of any commercial or financial relationships that could be construed as a potential conflict of interest.

## Publisher’s Note

All claims expressed in this article are solely those of the authors and do not necessarily represent those of their affiliated organizations, or those of the publisher, the editors and the reviewers. Any product that may be evaluated in this article, or claim that may be made by its manufacturer, is not guaranteed or endorsed by the publisher.
