# Supplementary material for: Implicit, Explicit, and Structural Barriers and Facilitators for Information and Communication Technology Access in Older Adults
Source: Front Psychol. 2022 May 17;13:874025. doi: 10.3389/fpsyg.2022.874025 (PMC9200138; doi:10.3389/fpsyg.2022.874025)
Supplement: Supplementary file 1 [file Table_1.DOCX]

Supplementary Material

1. **Interview guide (from Spanish translated to English)**

Presentation:

- May I call you by your first name?
- Where were you born? How many years have you been in Madrid? How old are you? What have been your jobs before?
- Neighborhood: Have you been living here a long time? / Do you associate with your neighbors of your building or the neighborhood? / Do you participate in any way in associations or activities in the neighborhood - e.g. IMERSE?
- Family: Do you have children? / Do you have a husband or wife? / Do you have siblings? / Do you see them very often? / How does your family support you in your day to day?
- Did you go to college- university- vocational training? / Can you read and write?
- Do you do physical exercise? Who do you do it with or alone?
- What is the biggest problem and the greatest facility that older people have in Spain?

Technology: use the phone and tablet in the meantime

- Do you like technologies? / Do you consider them useful? / Do you use them a lot and do you know how to use them? / What devices do you use the most, TV, radio, landline, etc.?
- Do you have a mobile phone? Do you know the difference between a Smartphone and a normal mobile?
- What brand of mobile do you use? / What company? / Who does the fee payment arrangements?
- How many years have you been using a mobile phone? / How often do you use it?
- Do you take your mobile outside?
- What do you use it for?
- Do you call very often? / Who do you normally call? / Do you send text messages or use WhatsApp?
- What reason leads you to use your mobile? / Feeling of security, relating to people, to monitor your health, as a reminder of birthdays or other events, to live more independently, etc.
- What mobile applications do you normally use? / Alarm, calendar, email, health apps, etc.?
- Do you know what social networks are? / Do you have WhatsApp, Facebook, twitter or any other social network? / What do you use it for?
- Do you play video games on your mobile?
- Do you use the google search engine?
- Do you read the press on your mobile?
- Do you listen to music on your mobile?
- Learning: Did someone help you start using it or did you learn by yourself? / Who in your family helped you? / Did they teach you how to use it in the store where you bought the phone?
- Prices: Do you consider the mobile phone a very expensive device? / Did you buy the mobile or was someone who bought it for you? / Who sold it to you was clear and honest about the price and use?
- Risks: do you feel that you lose privacy when using your mobile? / Do you feel that you isolate from the world or do you feel that it opens doors for you?
- Barriers: What difficulties do you have when using your mobile?
- Is the screen too small?
- Are the keys very small?
- Is the screen quality bad or good?
- Is the menu very complex?
- Do you feel anxious when using it or do you feel confident?
- Is the telephone manual understandable?
- Do your eyesight and your physical abilities allow you to use your mobile well?
- Do you think that when you started using your mobile it caused a sudden change in your day to day?
- Which apps do you struggle the most with?
- Do you find problems remembering passwords or PINs?
- Tablet: Do you have one of these devices? / Do you use this device very often? / Do you know what are the differences between a mobile and this? / Do you consider it more difficult to use than a mobile or easier? / What apps do you use on the tablet that you don't use on the mobile?

Conclusions

- Do you think that mobile phones and tablets should be better adapted to older people? / In what way?
- Would you prefer to use a Smartphone or a classic mobile?
  1. **Additional questions during COVID-19**
- How are you feeling during the outbreak and quarantine?
- What technologies are you using during this time?
- How did you find out about the applauses to the healthcare professionals?
- Who do you often talk with?
- Do you use WhatsApp, Facebook, etc. to stay informed about Coronavirus?
- Has your perception on technologies changed since the outbreak?
- Do you feel in this moment more protected by technologies?
- Have you tried any new app nowadays?
- How do you get in touch with your doctor?

1. **Situational analysis**

The following supplementary material is included to explain how we analyzed the data and how we came to use the structural, explicit, and implicit frameworks.

The non-human elements comprise ICT and are linked by a blue line. The explicit discourses built upon by the non-human actants are connected with a black line and the silent-symbolic elements with a pink line. The structural elements are associated with a purple line. The human elements, debates, spatial, and temporal factors are not associated per se but linked to particular ICT, socio-cultural and structural actants. The rest of authors reviewed the connections, and we perceived that social identities (light brown line) were not categories per se but they cut across other categories.

**
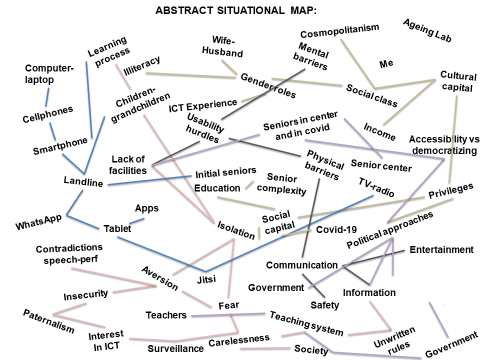
**

**Figure 1**: first messy map of the elements that constitute the research.
